# Effect of Plant Polyphenols on Adipokine Secretion from Human SGBS Adipocytes

**DOI:** 10.1155/2011/285618

**Published:** 2011-09-22

**Authors:** Christos S. Derdemezis, Dimitrios N. Kiortsis, Vasilis Tsimihodimos, Maria P. Petraki, Patra Vezyraki, Moses S. Elisaf, Alexandros D. Tselepis

**Affiliations:** ^1^Laboratory of Physiology, School of Medicine, University of Ioannina, 45110 Ioannina, Greece; ^2^Department of Internal Medicine, School of Medicine, University of Ioannina, 45110 Ioannina, Greece; ^3^Department of Chemistry, University of Ioannina, 45110 Ioannina, Greece

## Abstract

*Introduction*. Adipose tissue contributes to atherosclerosis with mechanisms related to adipokine secretion. Polyphenols may exhibit antiatherogenic properties. The aim of the study was to investigate the effects of three polyphenols, namely, quercetin, epigallocatechin gallate (EGCG), and resveratrol on adipokine secretion from cultured human adipocytes. *Methods*. Human SGBS adipocytes were treated with quercetin, EGCG, and resveratrol for 24 and 48 hours. Visfatin, leptin, and adiponectin were measured in the supernatant. *Results*. Visfatin secretion was inhibited by quercetin 10 **μ**M by 16% and 24% at 24 and 48 hours respectively. The corresponding changes for quercetin 25 **μ**M were 47% and 48%. Resveratrol 25 **μ**M reduced visfatin by 28% and 38% at 24 and 48 hours. EGCG did not have an effect on visfatin. None of tested polyphenols influenced leptin and adiponectin secretion. *Conclusion*. Quercetin and resveratrol significantly decreased visfatin secretion from SGBS adipocytes. This effect may contribute to their overall antiatherogenic properties.

## 1. Introduction

Obesity, defined as body mass index over 30 kg/m^2^, has emerged over the past thirty years as a major public health issue. The extent of the problem is ever-growing with incidence reaching epidemic dimensions, especially in Western societies. Obesity increases the risk of developing insulin resistance, diabetes mellitus, hypertension, and coronary heart disease and overall is associated with elevated morbidity and mortality [1]. Several explanations have been offered [2], yet one widely accepted is that adipose tissue serves more than mere neighboring organ insulator and triglyceride depot storage [3]. In fact, adipocytes produce and secrete a variety or proteins, collectively termed adipokines that exhibit important metabolic and inflammatory properties, [3]. Of particular interest are adipokines leptin, adiponectin and visfatin, each with unique properties.

Leptin, the primary adipokine, has a primary role in appetite suppression and downregulation of food intake [2, 3]. Thus, knock-out mice are massively obese. In humans though only few cases have been described, while the common form of obesity is presented with high leptin levels, indicating a condition of leptin resistance. Insulin resistance and diabetes are also associated with higher leptin levels. Moreover, leptin has been found to exhibit a role in lipoprotein and glucocorticoid metabolism, as well as in immune function. Similarly to leptin, adiponectin is mainly produced from adipose tissue, as the most abundant adipokine [2, 3]. Adiponectin levels are decreased in obesity, insulin resistance, and type 2 diabetes. It has been proposed that low circulating adiponectin levels are indicative of increased cardiovascular risk, since it exhibited antiatherogenic, insulin-sensitizing, and anti-inflammatory properties. Visfatin, initially identified as a growth factor for early B-cells, is mainly produced by visceral fat [4, 5]. It exerts insulin-mimetic effects in vivo and in vitro and is upregulated in obesity, metabolic syndrome, and diabetes. It is also implicated in dyslipidaemia, hypertension, and generally atherosclerotic-related diseases [4, 5]. We have recently shown that subjects with metabolic abnormalities that predispose to the development of cardiovascular disease exhibit increased serum visfatin concentrations [4, 5]. However, the pathophysiologic significance of this relationship remains to be elucidated. 

Combating obesity has been under scientific investigation for many years, and several strategies are being developed to manage this public health issue. Dietary modulation is considered first-treatment option. Plant-derived polyphenols, such as flavonoids and resveratrol, as part of human nutrition, have been put on test. Epidemiological and clinical evidence suggests a protective role against cardiovascular disease [6–8]. In addition, results from both animal and clinical studies are promising regarding antiobesity effects [9–13]. In the present study, we aim to investigate the direct effect of flavonoids quercetin, epigallocatechin gallate (EGCG), and resveratrol on the secretion of leptin, adiponectin, and visfatin from human adipocytes in culture.

## 2. Methods

Quercetin, EGCG, resveratrol, Dulbecco's modified Eagle's medium (DMEM)/Ham's F12, penicillin/streptomycin, biotin, pantothenate, L-glutamine, isobutylmethylxanthine (IBMX), dexamethasone, insulin, transferring triiodothyronine (T3), were purchased from Sigma (St. Louis, USA), fetal calf serum (FCS) from GIBCO (California, USA), and rosiglitazone (BRL49653) from Cayman Chemicals (Mich, USA). Simpson-Golabi-Behmel syndrome (SGBS) cells were maintained and differentiated into adipocytes as previously described [14]. In brief, cells were cultured in 24-well plates in DMEM/Ham's F12 supplemented with 10% FCS, penicillin/streptomycin, L-glutamine, 33 *μ*M biotin, and 17 *μ*M pantothenic acid and incubated at 37°C in a humidified atmosphere of 5% CO_2_. Cells were grown to confluence for 5 days. Subsequently, we initiated the adipocyte differentiation program. Medium was changed into serum-free DMEM/F12, containing penicillin/streptomycin, biotin, pantothenic acid, 0.01 mg/mL transferrin, 0.1 *μ*M cortisol, 200 pM T3, 20 nM human insulin, 0.25 *μ*M dexamethasone, 500 *μ*M IBMX, and 2 *μ*M rosiglitazone (differentiation medium). On day 4, medium was replaced by the differentiation medium lacking rosiglitazone, IBMX, and dexamethasone (medium 2) and cells were cultured for additional 11 days in medium 2, which was changed every 3 days [14].

The cell differentiation into adipocytes was confirmed by (1) microscopic observation with simultaneous oil red O staining (0.5% oil red O) [15]; (2) determination of the triglyceride (TG) content in the cell lysate with the GPO Trinder method [16]. To prepare the cell-lysate, adipocytes were washed 3 times with phosphate-buffered saline (PBS, pH 7.4) and then were lysed with 200 *μ*L of lysis buffer containing 0.1% Triton X in PBS. Cell lysate was further homogenized by sonication. The protein content of cell lysate was determined by the Lowry method [17]. 

The effect of polyphenols on adipokine secretion was studied on fully differentiated adipocytes, that is, at 15 days [14]. Three independent experiments were performed in duplicate: SGBS adipocytes were rinsed twice with PBS and then treated for 24 and 48 hours with medium 2, containing additionally quercetin, EGCG, or resveratrol. All polyphenols were dissolved in dimethyl sulfoxide (DMSO). The maximum DMSO concentration into the culture medium did not exceed 0.2% by volume. Secreted adipokines were determined in duplicate in the cell supernatant collected from each well. Leptin and adiponectin were measured using kits purchased from BioVendor (Czech Republic) and visfatin with a kit from Alpco (Salem, USA) according to manufacturer's protocol. Coefficient of variation for within and between kits was less than 7% for all three kits. Each concentration obtained was determined from the standard curve and after adjustment for protein lysate content of respective well. Results are reported as means with the standard error of the mean. Statistical analysis was performed using SPPS statistical package for Windows (SPSS Inc., Chicago, Ill, USA). Distribution was evaluated by Kolmogorov-Smirnov test, and comparisons of only two group means were performed by Student's *t*-test. Significance was set at *P* ≤ 0.05.

## 3. Results

We obtained fully differentiated mature adipocytes, 15 days after differentiation program induction, in accordance with previously published results [14]. Adipocyte differentiation was confirmed by microscopic observation of cells stained with oil red O (data not shown) and by quantitation of the cell TG content, expressed per *μ*g of cell protein. Before the initiation of the differentiation program, the TG concentration was lower than the detection limit of the method used in the present study (i.e., lower than 16 *μ*g/mL); however, 15-days differentiated mature adipocytes contained detectable amounts of TG having a TG to cell protein ratio of 1.14 ± 0.14 *μ*g/*μ*g (no difference was observed between various wells). Mature adipocytes secrete in the supernatant all three adipokines evaluated in the present study, 24 hours after changing the medium 2, whereas none of them was detected in the supernatant from cultured undifferentiated fibroblasts at day 4 (data not shown), thus further indicating functionality of adipocytes (leptin 98 ± 22 ng/mg cell protein, adiponectin 81 ± 23 ng/mg cell protein, visfatin 320 ± 70 ng/mg cell protein).

**Figure 1:**
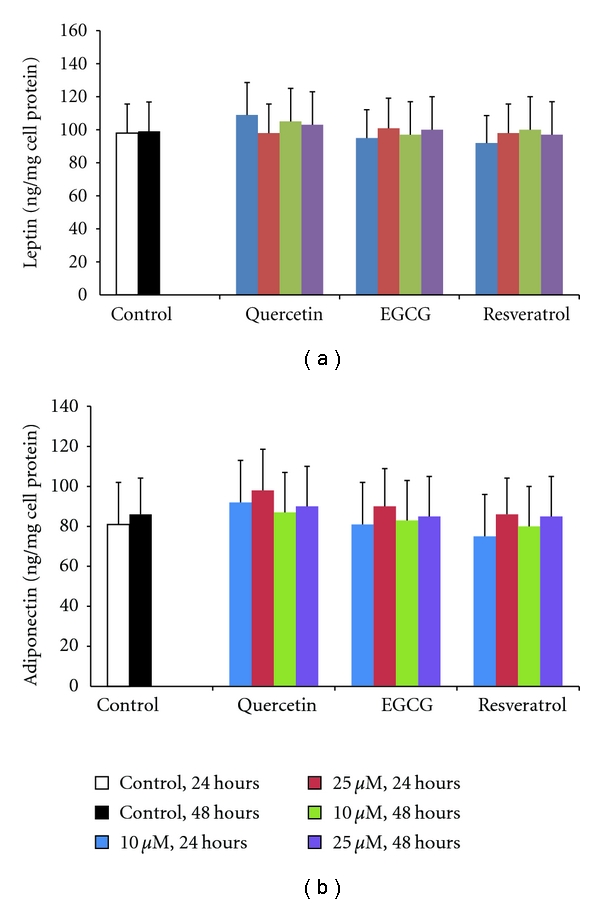
Polyphenols quercetin, epigallocatechin gallate (EGCG), and resveratrol do not affect leptin (a) and adiponectin (b) secretion by human mature SGBS adipocytes. Cells were cultured in medium and incubated with 10 or 25 *μ*M of quercetin, EGCG, and resveratrol for 24 and 48 hours. Leptin and adiponectin were determined in the cell supernatant by commercially available methods. Values represent the mean ± SD.

**Figure 2:**
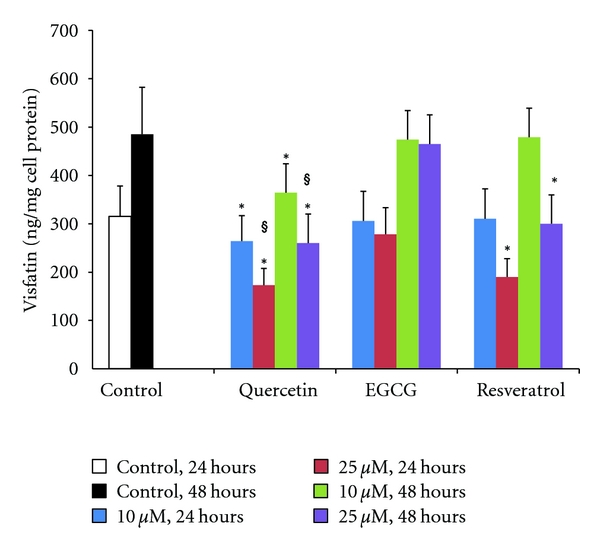
Effect of polyphenols quercetin, epigallocatechin gallate (EGCG), and resveratrol on visfatin secretion by human mature SGBS adipocytes at 24 and 48 hours. Cells were cultured in medium and incubated with 10 or 25 mM of quercetin, EGCG, and resveratrol for 24 and 48 hours. Visfatin levels were determined in the cell supernatant by commercially available methods. Values represent the mean ± SD. *P < 0.05 compared with corresponding control, ^§^ *P* < 0.05 compared with the value obtained at 10 *μ*M.

The effect of polyphenols used in the present study on adipokine secretion was studied as described in Materials and Methods. In preliminary experiments, we used polyphenol concentrations ranging from 10 to 50 *μ*M. However, at concentrations higher than 40 *μ*M, a precipitate was observed in the cell supernatant for all polyphenols, immediately after the substance addition. Hence, we evaluated their effect on adipokines' secretion at lower concentrations, that is, at 10 and 25 *μ*M. None of the three polyphenols tested influenced cell TG/protein ratio and leptin or adiponectin concentration in the cell supernatant at 24 hours and 48 hours (Figure 1). Similarly, EGCG had no effect on visfatin secretion at both time points. By contrast, incubation of adipocytes with quercetin resulted in a significant dose-dependent reduction of visfatin secretion (Figure 2). More specifically, quercetin 10 *μ*M reduced visfatin secretion by 16% and 25% at 24 and 48 hours, respectively (*P* < 0.05 versus control), whereas the corresponding changes for quercetin 25 *μ*M were 47% and 48% (*P* < 0.05 versus control and *P* < 0.05 versus 10 *μ*M in the same time points). Low concentration of resveratrol had no effect on visfatin secretion, while the 25 *μ*M of the substance decreased visfatin concentration by 28% and 38% at 24 and 48 hours, respectively, (*P* < 0.05 versus control for both comparisons). These changes were similar to those observed with the high dose of quercetin.

In order to evaluate whether the decrease in the visfatin concentration in the cell supernatant of cells treated with quercetin or resveratrol is due to the reduction in visfatin production, we determined the quercetin and resveratrol effects on both the cell-associated and the secreted visfatin levels. We measured intracellular and secreted visfatin levels in the presence of both polyphenols at both concentrations tested. Measuring intracellular visfatin in cell lysate yielded minimal amounts, in the order of hundredth relative to the total visfatin in the supernatant and without difference between wells (4.1 ± 0.9 ng visfatin/mg cell protein, p = NS between wells), suggesting that (1) in SGBS adipocytes, visfatin is a secretory protein and (2) quercetin and resveratrol reduced its production.

## 4. Discussion

Obesity is now considered a low-grade inflammatory state. Thus, it is supported that its management should not solely be targeted to weight loss, but also towards correction of metabolic imbalance of adipokines. In the present study, we aimed to investigate possible effect of three specific plant polyphenols on adipokine secretion from human adipocytes in culture. We selected three polyphenols which have been considered representative, more abundant in human nutrition and more promising in terms of positive effects in previous studies [18, 19]. Several investigators have evaluated the role of various flavonoids or resveratrol concerning their effects on preadipocyte differentiation, adipogenesis, and adipocyte apoptosis [20]. We focused on a different aspect of adipocyte metabolism, adipokine secretion. The usual model selected in previous studies is mouse strain 3T3-L1 or isolated rat adipocytes. However, in this study, we preferred to use the human SGBS cell line, because, given the species differences in adipose tissue metabolism [21], this cell line may more closely resemble human adipose tissue.

To the best of our knowledge, this is the first report on the polyphenol-induced modulation of visfatin secretion by human SGBS adipocytes. Interestingly, measuring visfatin in the lysate yielded minimal amounts, suggesting that the majority of total visfatin is excreted in the medium, in accordance with the findings of Revollo et al. in SGBS adipocytes [22]. Thus, we presume that polyphenols exert their effects on visfatin metabolism by modulating both the production and excretion of this molecule by SGBS adipocytes. In addition, our results support a substance-dependent differential effect of various polyphenols on visfatin secretion, a finding that may be related to the chemical structure of the polyphenols tested. Indeed, we found that quercetin more potently reduced visfatin secretion compared to resverartol (although these differences did not reach statistical significance), while the structurally different EGCG did not change visfatin secretion. Similarly, Chuang et al. [23] have recently demonstrated that quercetin more efficiently improves anti-inflammatory pathways and insulin resistance than resveratrol. Differential effects of different flavonoids on various aspects of adipose tissue metabolism have been reported elsewhere as well [24, 25]. Visfatin levels have been found increased in obesity, diabetes mellitus, hypertension, and cardiovascular disease. Although the nature of this relationship is still under investigation, the evidence points towards a proatherogenic role of this adipokine. In this regard, in vitro visfatin downregulation by polyphenols might be related to their antiatherogenic properties. 

The effect of polyphenols on leptin metabolism remains controversial. Thus, Tsuda et al. [26] showed that anthocyanin, another flavonoid, induced an increase in leptin levels in cell medium of isolated rat adipocytes. However, in our study, none of the polyphenols tested had an effect on leptin secretion. These differences can, at least in part, be attributed to that, by contrast to Tsuda et al., we did not incubate the cells with an inflammatory stimulant, such as TNF, that may increase baseline leptin levels. To make things more complicate, studies on rodents fed a diet rich in polyphenols or injected with EGCG revealed no change or even a decrease in serum leptin levels [27–30]. Certainly, comparison between cell cultures and animal studies is not direct.

We showed no change on adiponectin secretion in the medium with either treatment, a finding which suggests that, at least in mature adipocytes, the polyphenols studied have no effect. Interestingly, though, anthocyanin was reported to increase adiponectin gene expression and protein secretion in cultured rat adipocytes and tissue gene expression experiments [26]. However, apart from using different flavonoids than ours, the concentration used in this study was higher (4 to 10 fold). Yet, in a recent study by Cho et al. [25], some flavonoids, including quercetin and EGCG, decreased adiponectin in mouse 3T3-L1 adipocytes, whereas others increased it, thus further strengthening the view that various flavonoids may possess diverse effects and that adipose tissue metabolism may display important interspecies differences. In fact, even within the same model (3T3-L1) and between in vitro and in vivo studies, results can be contrasting [28, 31]. In human visceral adipocytes from obese women, resveratrol increased adiponectin [32]. Maybe, cultured SGBS adipocytes that are not treated with a proinflammatory stimulant exhibit a higher basal adiponectin excretion than (theoretically) more inflamed visceral adipocytes that may counteract inflammation after treatment with resveratrol. In addition, whole body metabolism of an antioxidant mix or green tea infusion including EGCG may impact indirectly on adipose tissue and adiponectin, for example, via improved liver function [33]. 

In conclusion, from the polyphenols studied, quercetin and resveratrol decreased visfatin secretion. No effect of the tested polyphenols on leptin and adiponectin secretion from human adipocytes in culture was noticed. To the best of our knowledge, this is the first time polyphenols are reported to modify visfatin secretion from cultured adipocytes. This finding might be a potentially beneficial effect of these compounds on adipose tissue and could contribute to their antiatherogenic actions. In the future, these polyphenols might be used pharmacologically for treating obesity-related metabolic abnormalities. Further research is required to verify our findings in cell cultures as well as in animal and human studies.
